# Random Forest Classification of Alcohol Use Disorder Using EEG Source Functional Connectivity, Neuropsychological Functioning, and Impulsivity Measures

**DOI:** 10.3390/bs10030062

**Published:** 2020-03-01

**Authors:** Chella Kamarajan, Babak A. Ardekani, Ashwini K. Pandey, David B. Chorlian, Sivan Kinreich, Gayathri Pandey, Jacquelyn L. Meyers, Jian Zhang, Weipeng Kuang, Arthur T. Stimus, Bernice Porjesz

**Affiliations:** 1 Henri Begleiter Neurodynamics Lab, Department of Psychiatry, SUNY Downstate Health Sciences University, Brooklyn, NY 11203, USA; ashwini.pandey@downstate.edu (A.K.P.); david.chorlian@downstate.edu (D.B.C.); Sivan.Kinreich@downstate.edu (S.K.); gayathri.pandey@downstate.edu (G.P.); jacquelyn.meyers@downstate.edu (J.L.M.); jian.zhang@downstate.edu (J.Z.); weipeng.kuang@downstate.edu (W.K.); arthur.stimus@downstate.edu (A.T.S.); bernice.porjesz@downstate.edu (B.P.); 2 Center for Biomedical Imaging and Neuromodulation, Nathan S. Kline Institute for Psychiatric Research, Orangeburg, NY 10962, USA; Babak.Ardekani@nki.rfmh.org; 3 Department of Psychiatry, NYU School of Medicine, New York, NY 10016, USA

**Keywords:** alcohol use disorder (AUD), functional connectivity, default mode network (DMN), resting state EEG, eLORETA, neuropsychological performance, Tower of London Test, Visual Span Test, impulsivity, Random Forest

## Abstract

Individuals with alcohol use disorder (AUD) manifest a variety of impairments that can be attributed to alterations in specific brain networks. The current study aims to identify features of EEG-based functional connectivity, neuropsychological performance, and impulsivity that can classify individuals with AUD (N = 30) from unaffected controls (CTL, N = 30) using random forest classification. The features included were: (i) EEG source functional connectivity (FC) of the default mode network (DMN) derived using eLORETA algorithm, (ii) neuropsychological scores from the Tower of London test (TOLT) and the visual span test (VST), and (iii) impulsivity factors from the Barratt impulsiveness scale (BIS). The random forest model achieved a classification accuracy of 80% and identified 29 FC connections (among 66 connections per frequency band), 3 neuropsychological variables from VST (total number of correctly performed trials in forward and backward sequences and average time for correct trials in forward sequence) and all four impulsivity scores (motor, non-planning, attentional, and total) as significantly contributing to classifying individuals as either AUD or CTL. Although there was a significant age difference between the groups, most of the top variables that contributed to the classification were not significantly correlated with age. The AUD group showed a predominant pattern of hyperconnectivity among 25 of 29 significant connections, indicating aberrant network functioning during resting state suggestive of neural hyperexcitability and impulsivity. Further, parahippocampal hyperconnectivity with other DMN regions was identified as a major hub region dysregulated in AUD (13 connections overall), possibly due to neural damage from chronic drinking, which may give rise to cognitive impairments, including memory deficits and blackouts. Furthermore, hypoconnectivity observed in four connections (prefrontal nodes connecting posterior right-hemispheric regions) may indicate a weaker or fractured prefrontal connectivity with other regions, which may be related to impaired higher cognitive functions. The AUD group also showed poorer memory performance on the VST task and increased impulsivity in all factors compared to controls. Features from all three domains had significant associations with one another. These results indicate that dysregulated neural connectivity across the DMN regions, especially relating to hyperconnected parahippocampal hub as well as hypoconnected prefrontal hub, may potentially represent neurophysiological biomarkers of AUD, while poor visual memory performance and heightened impulsivity may serve as cognitive-behavioral indices of AUD.

## 1. Introduction

Alcohol use disorder (AUD) is a chronic relapsing disorder characterized by an impulsive drive toward continued alcohol consumption despite negative consequences [1,2,3]. Individuals with AUD manifest a variety of structural and functional brain abnormalities [4,5,6,7]. For example, numerous functional magnetic resonance imaging (fMRI) studies in AUD have shown region-specific deficits in brain activation, mainly implicating the frontal lobes [8,9], during cognitive and affective processing, involving inhibitory control, executive functioning, memory, and reward processing [10,11]. These functional abnormalities during cognitive processing in AUD have been well-characterized by neurophysiological studies of electroencephalogram (EEG), event-related potentials/oscillations (ERP/ERO) [5,6,12], as well as by neuropsychological studies [7,13] and behavioral manifestations, such as impulsivity [14]. Structural MRI studies using diffusion tensor imaging (DTI) have shown abnormalities in whiter matter integrity and connectivity across several fiber tracts connecting different brain regions across cortical as well as subcortical structures, due to chronic alcohol exposure [4,15,16,17]. Recent studies have identified that neurostructural damage in AUD was significantly related to functional deficits in executive performance, which was in turn associated with microstructural changes in large-scale brain networks [18,19]. In summarizing brain changes due to chronic alcohol addiction, Sullivan and Pfefferbaum [20] stated that (i) alcoholism affects selective brain systems and circuits, and (ii) alcoholism causes “incomplete brain lesions” without destroying the neurons, leaving the potential for recovery. These findings and insights from the literature suggest the importance of understanding brain networks affected in AUD, and there is a fast-growing number of studies that attempt to elucidate network mechanism and dysfunction in AUD (e.g., [21,22,23,24,25,26,27,28,29,30]).

While these studies have identified specific deficits in neural, cognitive and behavioral domains in AUD, a distinct combination of characteristic features from multiple domains that can successfully classify individuals with AUD diagnosis from unaffected controls has not yet been done. In recent years, Machine Learning approaches have been commonly used to predict and/or classify various neuropsychiatric disorders and outcomes [31,32,33], including AUD [29,34,35]. Random forest (RF), introduced by Leo Breiman [36], is one of the widely used machine learning methods to classify individuals with a particular diagnosis from unaffected controls [37]. According to Sarica et al. [37], the RF method is more protective against overfitting, adaptive to highly non-linear data, and also credible for the data with outliers. Recently, Zhu et al. [29] applied the RF algorithm to successfully classify AUD subjects from control individuals using fMRI based resting state networks and concluded that machine-learning algorithms can serve as alternative techniques to quantify large-scale network differences across clinical groups and to identify of potential biomarkers for a specific diagnostic category. 

Brain electrophysiological measures, such as electroencephalogram (EEG) and event-related potentials/oscillations (ERP/ERO), remain the most valuable method to study the sensory, motor, and cognitive phenomena as they unfold, due to their excellent time resolution at the scale of milliseconds [12]. Although fMRI has higher spatial resolution than EEG, it is nevertheless an indirect measure of neural activity based on the brain’s metabolic activity and has poor temporal resolution owing to the biophysics of the hemodynamic response [38]. On the other hand, EEG records direct neural activity resulting from the excitatory and inhibitory signals of synchronously firing neurons [39]. While it is recognized that the plethora of neural processes that operate in a domain of tens of milliseconds will remain beyond the capabilities of the fMRI techniques [38], fast ongoing neurocognitive processes and their brain network dynamics can be investigated with EEG based measures. 

The utility of brain electrophysiological measures to understand neurocognitive dynamics in AUD have been well-documented [5,6,12,35,40,41]. However, until recently, the utility of these measures to understand brain network mechanisms in terms of neuroanatomical connections were beyond reach due to their poor spatial resolution. With the advent of modern techniques using source modeling of EEG signals in three-dimensional cortical voxels, it became possible to study brain connectivity across specific networks. One such method is the exact low-resolution brain electromagnetic tomography (eLORETA) [42], which has been commonly used to assess EEG-based functional connectivity, a measure of temporal synchrony between brain signals across different anatomical regions in healthy individuals (e.g., [43]) as well as in those with neuropsychiatric disorders (e.g., [44,45]).

Resting-state brain networks have been found to display relatively stable anatomical distributions and functional attributes of ongoing brain dynamics [46]. Among the seven major resting state networks described by Raichle [47], the default mode network (DMN) is the most well-characterized network. The DMN plays a central role in the intrinsic network properties and organization [48] and represents basic neural activity underlying self-referential thought, mentation, and introspection [49]. According to Molnar-Szakacs and Uddin [50], nodes of the DMN selectively interact with brain systems for embodiment and mentalizing, including the mirror neuron system, to produce appropriate cognitive mappings demanded by the ongoing mental or social context. DMN nodes also modulate cognitive processing during task-related activity [51], resulting in task-related suppression in the DMN regions [52]. Further, a lack of such suppression in DMN nodes during cognitive processing has been observed in psychopathology [53]. Therefore, studying DMN is important to understand ongoing mental processes as well as resting state brain dynamics in AUD individuals.

A growing number of studies have examined fMRI based resting state functional connectivity in AUD and found aberrations in neural communications across brain regions in several networks (e.g., [23,26,28,29]). Specific to DMN, Chanraud et al. [21] were the first to report compromised functional connectivity across the DMN nodes, showing that the posterior cingulate and cerebellar regions in alcoholics had lower synchronization (hypo-connectivity) compared to controls. Muller-Oehring et al. [26] reported that alcoholics showed DMN related aberrations of fMRI hypo-connectivity of posterior cingulate with right caudate region and hyper-connectivity of PCC with right occipital regions, further implicating the posterior cingulate region. In contrast, Kim et al. [28] reported that AUD individuals did not differ in fMRI FC of the DMN but manifested lower FC in the executive control network and the dorsal attention network. Zhu et al. [3] investigated multiple regions involving several networks and found that individuals with AUD exhibited within-network hyperconnectivity in DMN, salience, orbitofrontal cortex, left executive control and amygdala-striatum networks. Recently, in another fMRI FC study using random forest (RF), Zhu et al. [29] reported that executive control networks showed the strongest prediction accuracy among the within-network features, while the connectivity between DMN and reward network as well as between executive control network and reward network contributed more to the predictive accuracy in classifying AUD from controls. Recently, using the same groups of individuals as the current study, we have also reported aberrant fMRI based FC across the DMN regions in AUD individuals [54]. In sum, these fMRI based FC studies in resting state revealed aberrations in AUD in DMN and other resting state networks.

EEG measures in general represent direct neuronal activity with high time-resolution at the millisecond level, thereby detecting fast, ongoing neural processes underlying oscillatory dynamics [55,56]. The eLORETA, a linear, discrete, three-dimensional weighted minimal norm inverse solution method [42], enables the non-invasive examination of intra-cortical interactions with interpretable spatial resolution [57]. Specifically, the lagged phase synchronization of the eLORETA is predominantly used to assess functional connectivity (e.g., [45,58]), as it represents physiological (neural) information and is minimally affected by the low spatial resolution [42]. To our knowledge, the only study that is available on resting state EEG source (eLORETA) connectivity on alcoholism [59] has examined a limited number of AUD patients (N = 11) who had excessive craving and withdrawal symptoms and found a dense array of hyperconnectivity (i.e., increased FC) in theta band across the regions of reward and executive processing networks. Thus, although neurophysiological markers of AUD at the finer time scale of neural communication can uncover subtle, sensitive, real-time, ongoing neurocognitive dynamics [60,61,62], they remain largely unknown due to paucity of studies. Thus, the current study is the first to fill this gap to examine eLORETA-based DMN FC features to classify individuals with AUD from unaffected controls by applying the RF model to a relatively larger sample of individuals with AUD (N = 30). Further, in order to improve the prediction accuracy of the RF model, in addition to the neural connectivity features, it is important to include other characteristic features of AUD, such as neurocognitive performance and impulsivity, as the individuals with AUD are also known to manifest a range of neuropsychological impairments [63,64] and heightened impulsivity [65]. Therefore, the RF model in the current study will use features from all three domains (i.e., EEG FC, neurocognition, and impulsivity). 

Taken together, studies from multiple domains of neural features have established both structural and functional abnormalities in individuals with AUD. The findings from these studies converge to emphasize the importance of understanding brain networks affected in AUD, and therefore numerous recent studies have attempted to elucidate network mechanisms and dysfunctions underlying AUD by studying functional connectivity. However, there is a critical lack of studies examining functional connectivity using neurophysiological sources, and the current study attempts to fill the gap in knowledge by examining EEG source functional connectivity of the DMN, the most studied resting network, in addition to identifying other relevant features that are characteristic of AUD (e.g., measures of neuropsychological function and impulsivity). The a priori hypothesis of the current study is that individuals with AUD will show distinct and differential patterns in DMN functional connectivity, neuropsychological measures, and impulsivity factors, compared to unaffected controls, as elicited by a classification algorithm such as Random Forest [36]. We expect that the RF algorithm will identify specific connections in different frequency bands across the DMN nodes, especially in frontal regions and their long-range connections with other regions, together with neuropsychological and impulsivity features that are significantly important to classify AUD subjects from unaffected controls. 

## 2. Materials and Methods

### 2.1. Participants

The demographic and clinical characteristics of the sample are presented in Table 1, and a detailed sample description is available in Pandey et al. [16] and Kamarajan et al. [54]. Briefly, the sample comprised thirty male participants with AUD [mean age (standard deviation, SD) = 41.42 (7.31) years] and thirty unaffected male controls [mean age (SD) = 27.44 (4.74) years]. Individuals for the control group (CTL) were recruited through advertisements and screened to exclude any personal or family history of major medical, psychiatric, or substance-related disorders. Participants with AUD (DSM-IV alcohol dependence criteria) were recruited from alcohol treatment centers in and around New York City after they had been detoxified and were abstinent for at least 30 days prior to testing. Participants were not in withdrawal at the time of testing. A modified version of the semi-structured assessment of genetics of alcoholism (SSAGA) [66] was administered to assess alcohol/substance use and related co-existing disorders and family history of these disorders. The vast majority of subjects were right-handed, with only a few who were either left-handed (5 in the AUD group and 2 in the CTL group) or bi-dexterous (2 in the AUD group and 1 in the CTL group). EEG, clinical and psychometric data were collected at the SUNY Downstate Health Sciences University. Individuals with hearing/visual impairment, a history of head injury or moderate and severe cognitive deficits (< 21 on the mini-mental state examination (MMSE) [67] were also excluded from the study. Informed consent was obtained from the participants and the research protocol was approved by the Institutional Review Board (SUNY IRB approval ID: 266893).

### 2.2. Neuropsychological Assessment

#### 2.2.1. Tower of London Test (TOLT)

The Tower of London Test (TOLT) [68], part of the Colorado assessment tests for cognitive and neuropsychological assessment [69], assesses planning and problem-solving ability of the executive functions. During the test, participants required to solve a set of puzzles with graded difficulty levels by arranging the color beads one at a time from a starting position to a desired goal position in as few moves as possible. The test consisted of 3 puzzle types with 3, 4, and 5 colored beads placed on the same number of pegs, with 7 trials per puzzle type and a total of 21 trials. Five performance measures from the sum total of all puzzle types were used in the analysis: (i) excess moves (additional moves beyond the minimum moves required to solve the puzzle); (ii) average pickup time (initial thinking/planning time spent until picking up the first bead to solve the puzzle); (iii) average total time (total thinking/planning time to solve the problem in each puzzle type); (iv) total trial time (total performance/execution time spent on all trials within each puzzle type); and (v) average trial time (mean performance/execution time across trials per puzzle type).

#### 2.2.2. Visual Span Test

The Visual Span Test (VST) [70,71], also part of the Colorado assessment tests, was used to assess visuospatial memory span from the forward condition and working memory from the backward condition. In this test, 8 randomly arranged squares were displayed on the screen, and some of the squares (i.e., 2–8 squares), depending on the span level being assessed, flashed in a predetermined sequence. The test consisted of 14 trials in each condition, as each span level was administered twice. Using mouse clicks, the subjects were required to repeat the sequence in the same order during the forward condition; the subjects were required to repeat the sequence in reverse order (starting from the last square) during the backward condition. A total of 8 scores were recorded (4 per condition): (i) total correct trials (total number of correctly performed trials); (ii) span (maximum sequence-length achieved); (iii) total average time (sum of the mean time taken across all trials performed); and (iv) total correct average time (sum of the mean time taken across all trials correctly performed).

### 2.3. Impulsivity Scores

The Barratt Impulsiveness Scale—Version 11 (BIS-11) [72], a 30-item self-administered questionnaire, was used to assess impulsivity. The BIS has excellent psychometric properties [73], and contains three factor scores, viz., motor impulsivity (BIS_MI), non-planning (BIS_NP), and attentional impulsivity (BIS_AI), and a total score (BIS_Tot).

### 2.4. EEG Data Acquisition and Preprocessing 

EEG was recorded during awake, resting state in all participants as they were seated on a comfortable couch with eyes-closed in a dimly lit, sound-attenuated RF-shielded booth (Industrial Acoustics, Inc., Bronx, NY, USA). A 61-channel electrode cap (Electro-Cap International, Inc., Eaton, OH, USA) based on the Extended 10–20 System [74,75,76,77] was used. The reference electrode was at the tip of the nose, and the ground electrode was placed at the forehead. The electrooculogram (EOG) was recorded by a supraorbital vertical electrode and by a horizontal electrode on the external canthus of the left eye. Electrode impedances were maintained below 5 kΩ. Electrical activity was amplified 10,000 times using SynAmps2 amplifiers (Compumedics, Charlotte, NC) and was recorded continuously over a bandwidth between near-DC (0 Hz) and 100.0 Hz on a Neuroscan system (Versions 4.3–4.5; Compumedics USA, Charlotte, NC) at a sampling rate of 500 or 512 Hz, based on the version of the Neuroscan collection system used (resampling was performed at 256 Hz—see below). The preprocessing was performed using custom scripts in Matlab (The MathWorks, Inc., Natick, MA). The following steps were performed on the entire EEG sweep: (i) data points were resampled to 256 Hz for harmonizing different sampling rates; (ii) bandpass filtering at 0.05–50 Hz to keep only the frequency range of interest; (iii) waveforms were “detrended” to remove upward/downward trending; and (iv) “de-meaning” was done by subtracting the gross mean from each data point in order to align the waveforms close to the zero-amplitude baseline. Then, the continuous data was segmented into 2 second epochs. Another batch of preprocessing steps were performed on each of the epochs: (i) detrending; (ii) baseline alignment by subtracting epoch mean from each data point; (iii) interpolation of missing data or “flat” channels by computing mean of surrounding nearest channels; (iv) removal of epochs with DC shift/drift involving voltage steps higher than 75 mV between any two adjacent sampling points; and (v) removal of possible EOG contaminated epochs if any data point was beyond the threshold of ± 100 μV or if the difference between lowest and highest amplitude within the epoch was 200 μV. Artifact free 50 random epochs were selected for each participant in each group for the functional connectivity analysis.

### 2.5. DMN Seed Regions and FC Calculations

**Figure 1:**
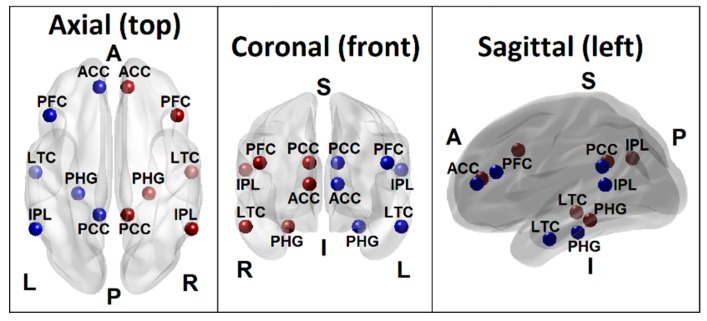
Seed regions of the default mode network (DMN) consisting of 6 regions in the left hemisphere (blue beads) and 6 homologous regions in the right hemisphere (red beads) as listed in Table 2. Axial (top), coronal (front), and sagittal (left) views are shown. [PCC–Posterior cingulate cortex; ACC–Anterior cingulate cortex; IPL–Inferior parietal lobule; PFC–Prefrontal cortex; LTC–Lateral temporal cortex; PHG–Parahippocampal gyrus; L–Left; R–Right; A–Anterior; P–Posterior; S–Superior; I–Inferior].

The DMN regions analyzed in the study are posterior cingulate cortex (PCC), anterior cingulate cortex (ACC), inferior parietal cortex (IPL), prefrontal cortices (PFC), lateral temporal cortex (LTC), and hippocampal formation (HCF) (Table 2 and Figure 1), in line with the fc-MRI and fc-EEG studies [58,78,79,80]. More information about the DMN seed selection is described in our recent publication with fc-MRI in the same sample as the current study [54]. Each seed region contained the voxels within 10 mm radius from the peak/centroid point of the region. The ROI-to-ROI connectivity [81], the most commonly used method to derive FC across brain regions [82], was computed using the exact low resolution electromagnetic tomography software (eLORETA) software [42], as described in the next section. 

### 2.6. EEG Based Functional Connectivity Analysis in eLORETA

The eLORETA is a signal processing software package designed for localizing the neuronal electrical activity in the brain, and also involves several methods to analyze EEG source data (current density) in 3-dimensional neuroanatomical space with 6239 voxels at 5-mm^3^ spatial resolution [42]. The eLORETA algorithm is an inverse solution for the scalp recorded EEG signals, using weighted minimum norm inverse solution [42]. The localization accuracy with regard to brain sources of the scalp EEG signals was achieved using specific weights in the eLORETA algorithm [83]. The lead field, which determines how the electrical activity recorded at scalp electrodes is reflective of the actual sources in the brain [84], was derived using a realistic volume conductor head model [85] based on the standardized MRI brain atlas (template) from the Montreal Neurologic Institute (MNI152) [86]. The eLORETA algorithm estimates the neuronal electrical activity from the EEG scalp measurements using the lead field and head model, using a three-dimensional solution space restricted to cortical gray matter and electrode coordinates [87]. At each voxel in the cortical grey matter, a 3-component vector time series is computed, corresponding to the current density vector with dipole moments along axes X, Y, and Z [88]. The values in individual voxel reflect the log-transformed fraction of total power across all 6239 voxels that covers the cortical regions of the brain, separately for specific frequencies [59]. While the original LORETA method used weighted minimum norm solution combined depth weighting and Laplacian smoothing, the latest version eLORETA uses optimal weights to achieve localization accuracy [89]. Further technical details of the eLORETA method are available elsewhere [42,88,89]. Inter-subject variability is minimized by the normalized eLORETA solutions, in which current density across voxels are averaged to a unit scale, rendering EEG power density in a Gaussian distribution [90]. In a recent study comparing eLORETA with other inverse methods, Halder et al. [91] found that while all these methods were successful in source identification if false positives were ignored, eLORETA was much superior even when false positives were accounted for. Localization capabilities and concordance of LORETA based methods have been reported by multimodal imaging studies of fMRI [92,93], structural MRI [94], and positron emission tomography (PET) [95,96], including studies with intracranial recordings in humans [97]. A growing number of studies are using eLORETA methods to examine current density activations and functional connectivity across brain regions to understand neurocognitive functioning and abnormalities (e.g., [43,44,45,57,58,98,99,100]).

Functional connectivity between intra-cortical sources is computed using lagged phase synchronization, which is less susceptible to volume conduction artifacts [88]. As explained by Canuet et al. [83], lagged phase synchronization represents nonlinear functional connectivity between any two signals reflecting phase similarity or synchrony. This measure is calculated for each frequency band as a normalized Fourier transform. Further, this method does not have artifactual zero-lag contribution (noise) but contains only the physiological signals of interest, because the instantaneous component of the total connectivity which is considered to be the confounding factor of volume conduction is statistically partialled out in this method [83]. This lagged phase coherence between two brain regions reflects neuronal communication between these regions, reflecting coherent, synchronous oscillatory activity between the sources [59]. According to Pascual-Marqui [88], the instantaneous coherence is the real part of the complex valued coherency and the imaginary part of the complex valued coherency is more affected by instantaneous dependence, whereas the lagged coherence, which is employed in eLORETA, is devoid of the confounding effect of instantaneous dependence due to volume conduction and low spatial resolution. In the current study, the eLORETA was used to extract time-series of current density for the different regions of interests (ROIs) and to compute ROI-to-ROI functional connectivity coefficients across the DMN seed regions (Figure 1 and Table 2) using linear lagged coherence across the seed regions on the preprocessed EEG segments for the custom frequency bands: delta (1–3 Hz), theta (4–7 Hz), alpha (8–12 Hz), beta (13–29 Hz), and gamma (30–40 Hz).

### 2.7. RF Classification Model and Parameters

The RF classification analysis was performed using R-packages “randomForest” [101], “caret” [102], and “randomForestExplainer” [103]. A RF classifier consists of collection of tree-structured classifiers where each tree casts a unit vote for a class/group for each set of predictor variables [36]. A growing number of studies in computational biology are using RF because of several advantages of the method. According to Qi [104], the RF method is not only nonparametric, but is interpretable and efficient. Further, the RF method can be applied to data with small sample size, multi-dimensional variables, and multiple layers/levels without compromising its prediction accuracy [104]. In a large-scale benchmark experiment, the RF algorithm was found to perform better than logistic regression in terms of prediction accuracy [105]. The two main parameters of the RF algorithm are the number of trees in the ensemble and the number of variables randomly selected for the splitting decision at each node. Two levels of randomness are used by the RF to construct the ensemble of trees: first, the model trains itself using a training data for creating each tree based on bootstrap aggregating (bagging). At the second level, the algorithm randomly selects a subset of features to split at each node while growing a decision tree for group classification. In order to maximize the classification accuracy (by reducing the errors or impurity), only a single best feature (variable) among a random subset of features is selected at each internal node. This process is recursively repeated until one of the three conditions is met: (i) the tree has either reached a specified depth, (ii) the number of samples in a node becomes lower than the set threshold, and (iii) when all the samples are grouped into the same category [106]. Some of the important concepts and parameters of RF classification method are listed in Box A1 (see Appendix A).

The RF classification model included 66 DMN connections for each of the 5 frequency bands, 13 neuropsychological scores, and 4 BIS scores as features, while the group status (AUD and CTL) served as the outcome variable. The training data consisted of full sample for identifying significant features for classifying the groups. To compute prediction error and classification accuracy, we used the out-of-bag (OOB) error estimate, which represents classification error obtained from the out of bag sample that were not part of the bootstrap sample used in growing the forests. In RF model, cross-validation in a separate test sample is not required, as it is estimated internally in the algorithm [107]. During each iteration of constructing a decision tree, only about two-thirds of the bootstrap sample from the training data is used and about one-third of the sample is left out during each bootstrap process, which is called the out-of-bag (OOB) sample. The classification error calculated from this sample is called the OOB error score. The aggregate of OOB scores from all decision trees will provide the ensemble OOB error rate (i.e., classification error) as well as the accuracy rate for the RF model. Thus, the OOB score provides a validation for the RF model. In the model, the maximum number of trees (‘ntree’) was set at 500. The optimal number of features analyzed at each node (‘Mtry’) was estimated to be 21 (using the ‘tuneRF’ function) and was used in the classifier algorithm. The final list of variables that significantly contributed for the classification was tabulated, and a 3-dimensional connectivity map of top significant DMN connections within a brain anatomical template was created using custom Matlab scripts. 

## 3. Results

### 3.1. RF Classification

#### 3.1.1. Classification Accuracy and Top Significant Variables

**Figure 2:**
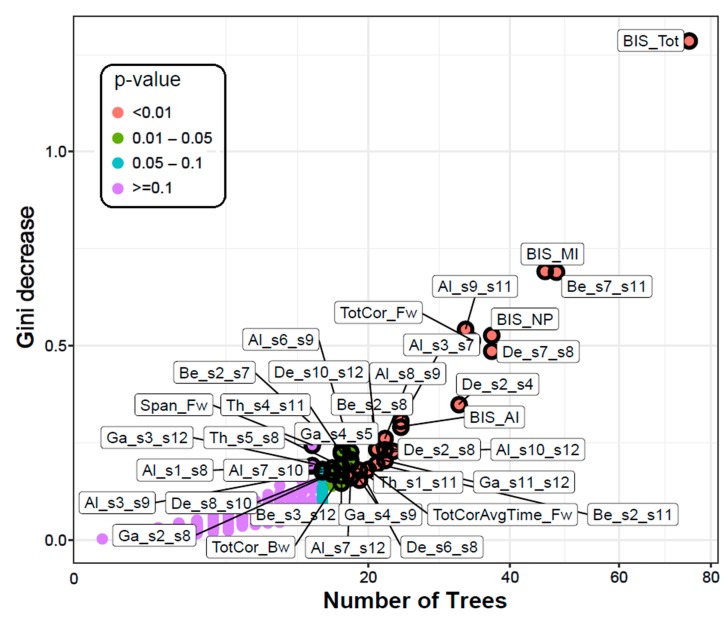
The multi-way importance plot showing the top significant variables (labelled and marked with black circles) that contributed to the classification of alcohol use disorder from control individuals based on the measures *Gini* *decrease, number of trees, and p-value*. All 4 impulsivity scores, 29 FC connections, and 3 neuropsychological variables were significant (circled and labelled red and gray dots). Impulsivity scores stood on top in the importance list. Note that the variables that were not significant or important (purple dots) are not highlighted. [Abbreviations in the FC variable labels: BIS–Barratt Impulsivity Scale; MI–Motor impulsivity; NP–Non-planning; AI–Attentional impulsivity; Tot–Total; Span_Fw–Span forward; TotCor_Fw–Total correct forward; TotCorAvgTime_Fw–Total correct average time forward; TotCor_Bw–Total correct backward; De–Delta; Th–Theta; Al–Alpha; Be–Beta; Ga–Gamma; s1-s12–default mode network (DMN) seeds 1-12 as listed in Table 2].

**Figure 3:**
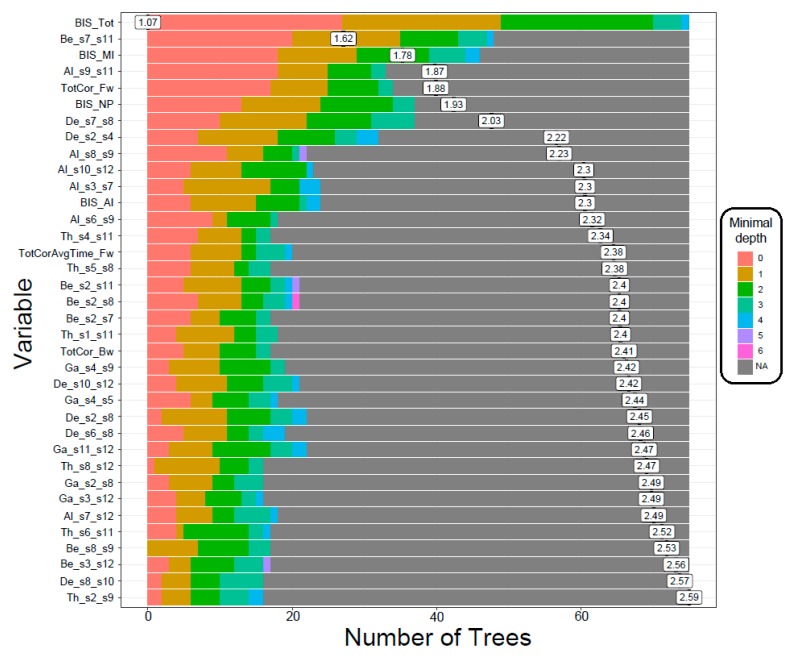
The distribution of minimal depth among the trees of the forest for the significant variables is shown in different colors for each level of minimal depth. The mean minimal depth in the distribution for each variable is marked by a vertical black bar overlapped by a value label inside a box. Lower mean minimal depth of a functional connectivity variable represents higher number of observations (participants) categorized in a specific group on the basis of the variable (i.e., better classification). The top significant variables (29 functional connectivity connections, 3 neuropsychological scores and all 4 impulsivity scores) followed the same rank in the plot as in Table 3, which is ordered based on *p*-values. [Abbreviations in the FC variable labels: De–Delta; Th–Theta; Al–Alpha; Be–Beta; Ga–Gamma; s1-s12–default mode network (DMN) seeds 1-12 as listed in Table 2].

**Figure 4:**
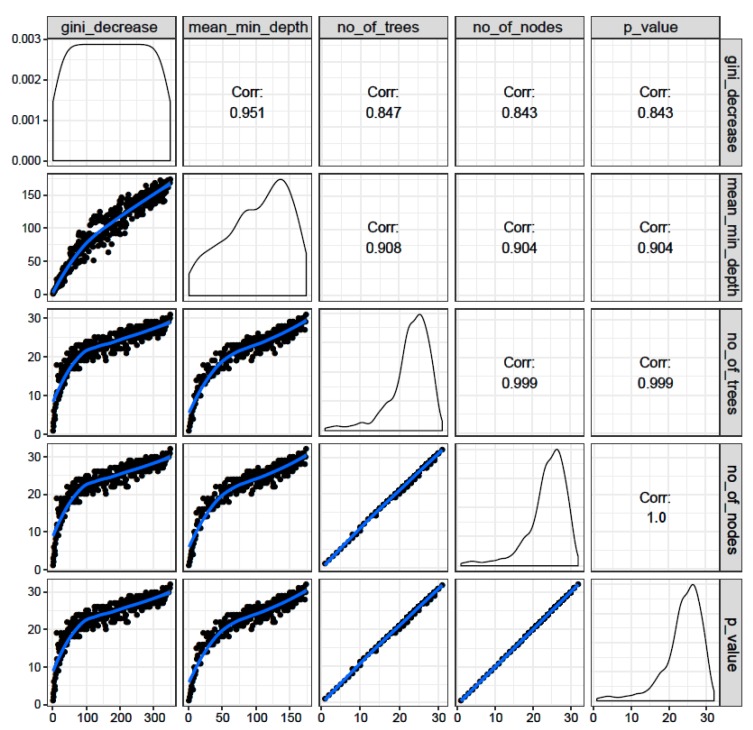
Illustration of rankings of variables based on any of the two Random Forest (RF) parameters of importance (panels in the lower triangle of the grid showing distribution of rankings of all predictor variables with black dots along a blue trend line) as well as correlation coefficient across rankings of any two parameters (panels in the upper triangle of the grid). It is shown that all RF parameters of importance were found to have very high correlations among each other, suggesting high reliability of each of these parameters to rank the importance of variables for group classification.

**Figure 5:**
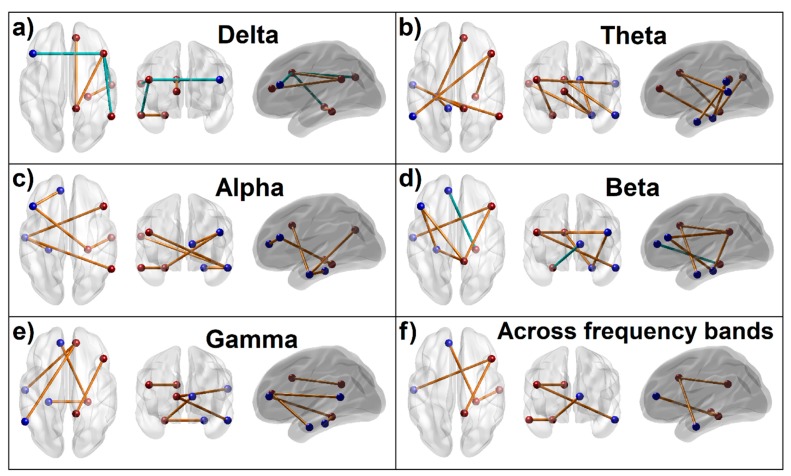
Panels (a–e): Significant default mode network (DMN) connections within each frequency band, which contributed to the Random Forest classification of alcohol use disorder (AUD) from control (CTL) individuals based on the importance parameters, viz., *p*-value (*p* < 0.05), number of trees, and Gini index as listed in Table 3. Panel (f): Common connections across different frequency bands. The three images within each panel represent three views, respectively: axial or top view, coronal or front view, and sagittal or left view. The blue and red beads represent left and right-sided DMN nodes/seeds respectively, and the connections represented in orange/red and cyan/blue lines indicate hyper- and hypo-connectivity, respectively, in AUD compared to CTL group. Note that one of the four common connections, L.ACC–R.PHG, although shown here in orange/red (panel f), showed hypoconnectivity in beta band (panel d) and hyperconnectivity in gamma bands (panel e).

The RF correctly classified 24 out of 30 subjects in each group with an accuracy rate of 80%. The model identified 29 FC connections (across multiple DMN nodes representing all frequency bands), three neuropsychological variables from the VST (total number of correctly performed trials in forward and backward sequences and average time of correct trials in forward sequence) and all 4 impulsivity scores (motor, non-planning, attentional, and total) as significantly contributing to classifying individuals into either AUD or CTL group (Table 3 and Figure 2 and Figure 3) based on specific RF parameters (Figure 4). The significant FC connections revealed that AUD group showed a predominant pattern of hyperconnectivity (i.e., increased FC) in all frequency bands (25 out of 29 connections), and only four connections with hypoconnectivity (three in delta and one in beta band) (Figure 5). Interestingly, 13 of the 29 connections (45%) were bilateral hippocampal (PHG) connections with other DMN nodes (bilateral PCC, bilateral ACC, R.IPL, bilateral PFC, bilateral LTC), in which AUD showed hyperconnectivity in 12 connections across all frequency bands, and hypoconnectivity (i.e., decreased FC) across L.ACC–R.PHG in beta band. Four of the 29 significant connections were common across frequency bands (i.e., represented in more than one frequency bands excepting the theta band), in which AUD showed either hyper- or hypo-connectivity than CTL group: (i) R.PCC–R.PFC (hyperconnectivity in delta, beta, and gamma bands); (ii) R.PFC–L.LTC (hyperconnectivity in alpha and beta bands); (iii) R.LTC–R.PHG (hyperconnectivity in delta and alpha bands); and (iv) L.ACC–R.PHG (hypoconnectivity in beta band and hyperconnectivity in gamma band). AUD individuals also showed increased impulsivity in all categories and poor neuropsychological performance in visuo-spatial working memory (i.e., lower memory span for both forward and backward trials, and increased time taken during forward trials).

#### 3.1.2. Multi-Way Importance

The top significant variables were also shown in a multi-way importance plot based on the RF importance measures *Gini decrease, number of trees, and p-value* (Figure 2). A variable is deemed significant if that variable is used for splitting more often than at random. As listed in Table 3, the variables that were found to be important for group classification are: 29 FC connections (across multiple DMN nodes representing all frequency bands), three neuropsychological variables (total number of correctly performed trials in forward and backward sequences and average time of correct trials in forward sequence), and all four impulsivity scores (motor, non-planning, attentional, and total).

#### 3.1.3. Distribution of Minimal Depth

The distribution of minimal depth among the trees of the forest for the top significant variables is shown in Figure 3. The minimal depth of a variable represents the depth of the node which splits on that variable and is the closest to the root of the decision tree. The lower mean minimal depth of a variable represents higher number of observations (participants) categorized in a specific group on the basis of that variable (i.e., better classification). The order/rank of the top significant variables (29 FC connections, three neuropsychological scores and all four impulsivity scores) followed the same pattern (as in Table 3) in the minimal depth plot, which is based on minimal depth and the number of trees. 

#### 3.1.4. Correlations among Rankings of Different RF Parameters

The correlations among rankings of different RF parameters are shown in Figure 4. The correlations across any two parameters were very high (*r* = 0.843–1.0), suggesting that all the RF parameters ranked the variables in a similar order, and that these parameters were highly reliable to classify the groups. 

#### 3.1.5. Connectivity Mapping of Significant FC Connections

The 3-D brain connectivity map of all significant FC connections (*p* < 0.05), which contributed to group classification, is shown in Figure 5. Results showed that 29 FC connections, involving all 12 seeds and all five frequency bands, were significant at the *p* < 0.05 threshold. The distribution of the connections across the frequency bands were: The nodes of these 29 connections were predominantly connecting to right hemisphere (33 right vs. 25 left). Four of the 29 connections represented more than once across different frequencies (Figure 5, panel f): (i) hyperconnectivity across R.PCC–R.PFC in delta, beta, and gamma bands, (ii) hyperconnectivity across R.PFC–L.LTC in alpha and beta bands, (iii) hyperconnectivity across R.LTC–R.PHG in delta and alpha bands, and (iv) hyperconnectivity across L.ACC–R.PHG in beta band and hypoconnectivity of the same in gamma band (Figure 5, panel-f). While the AUD group showed a predominant pattern of hyperconnectivity, except four prefrontal connections that showed hypoconnectivity (L.PFC–R.PFC, R.PFC–R.LTC, R.PFC–R.IPL in delta band and L.ACC–R.PHG in beta band) (Figure 5). Among these 29 significant connections, bilateral hippocampal (PHG) connections (13 out of 29) with other DMN nodes was a predominant feature with hyperconnectivity in all but one of its connections (L.ACC–R.PHG) in beta band. 

**Figure 6:**
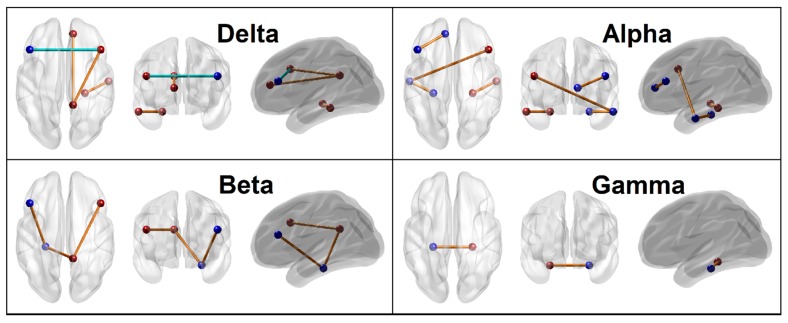
Significant default mode network (DMN) connections based on a stricter threshold (*p* ≤ 0.001) are shown. The three images within each panel representing specific frequency represent these three views, respectively: axial or top view, coronal or front view, and sagittal or left view. The blue and red beads represent left and right-sided DMN nodes/seeds respectively, and the connections represented in orange and cyan lines indicate hyper- and hypo-connectivity, respectively, in alcohol use disorder (AUD) compared to control group.

A stricter threshold of *p* ≤ 0.001 restricted the number of significant connections to 12 (from the original 29), which represented all except theta band (Figure 6). Out of this top 12 connections, six hippocampal connections showed hyperconnectivity with other DMN regions (R.PHG–R.LTC in delta band, L.PHG–L.LTC and R.PHG–R.LTC in alpha band, L.PHG–L.PFC and L.PHG–R.PCC in beta band, and L.PHG–R.PHG in gamma band). Further, AUD individuals also showed hypoconnectivity across prefrontal cortices and right hemispheric regions in the delta band.

### 3.2. Correlations between Significant Variables and Age

Since age difference across the groups was statistically significant (*p* < 0.001), the association of age with important predictor variables were evaluated within each group using bivariate Pearson correlation and in the total sample using partial correlation adjusted for group effect (Table 4) as an exploratory (descriptive) analysis. It was found that the association of age with the top variables were neither robustly significant nor consistent across these variables. One single connection (R.PCC–L.PHG) showed significant correlation with age in total sample as well as within each group (but did not survive Bonferroni correction), while the vast majority of the significant variables had no association with age. 

### 3.3. Correlations among the Top Significant Variables

**Figure 7:**
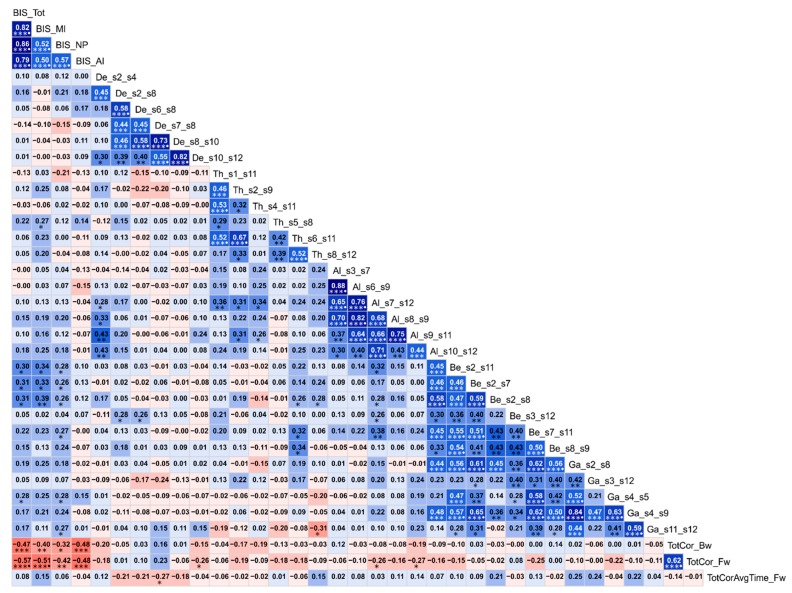
Correlation matrix showing associations among the top significant variables based on explorative (descriptive) correlational analysis for the interpretative purpose. Values within each cell represents bivariate Pearson correlation between the variable on its vertical axis and the variable on its horizontal axis. Correlation values are color coded [red/pink shades represent negative r-values, blue/cyan shades indicate positive r-values, darker color represent higher magnitude] and significant correlations (before Bonferroni correction) have been marked with asterisks [*p < 0.05; **p < 0.01; and ***p < 0.001]. The correlations that survived Bonferroni correction (*r* ≥ 0.50 and *p* < 0.00005) have been marked with a filled circle at the end of triple asterisks (***^●^). Abbreviations in the variable labels: De–Delta; Th–Theta; Al–Alpha; Be–Beta; Ga–Gamma; s1-s12–default mode network seeds 1-12 as listed in Table 2.

Exploratory (descriptive) analysis of correlations among top significant variables are shown in Figure 7. BIS impulsivity scores showed significant positive correlations among themselves while showing significant negative correlations with two of the neuropsychological scores of the VST, viz., total correct scores during forward and backward trials, suggesting that those with high impulsivity showed poorer neuropsychological performance. FC variables showed significant positive correlations with BIS scores in several frequency bands: one theta connection [s5–s8 (L.IPL–R.PFC)], four beta connections [s2–s11 (R.PCC–L.PHG), s2–s7 (R.PCC–L.PFC), s2–s8 (R.PCC–R.PFC), and s7–s11 (L.PFC–L.PHG)], and two gamma connections [s4–s5 (R.ACC–L.IPL) and s11–s12 (L.PHG–R.PHG)]. Interestingly, all these connections showed hyperconnectivity in AUD subjects who also displayed increased impulsivity than the CTL group, suggesting that hyperconnectivity was associated with increased impulsivity and that both phenomena were observed in AUD individuals. Further, significant correlations across neuropsychological scores and four FC connections were observed. A bilateral prefrontal delta connection (L.PFC–R.PFC), which had hypoconnectivity in AUD, was negatively correlated with average time of memory processing, suggesting that higher FC score (hyperconnectivity) was associated less average time or faster memory processing, while AUD showed an opposite pattern of hypoconnectivity across prefrontal nodes and slower memory processing. In the other three connections, viz., R.LTC–R.PHG (delta), L.PFC–R.PHG (delta), L.LTC–L.PHG (alpha), which were hyperconnected in AUD, had significant negative correlations (*p* < 0.05) with with total correct score during forward trials, suggesting that hyperconnectivity in these connections was associated with poor memory performance, as observed in AUD individuals. Furthermore, FC connections had significant positive correlations with each other within the same frequency as well as across different frequencies (theta–alpha, alpha–beta, and beta–gamma band). A few other isolated correlations across FC connections of different frequencies were also observed: (i) positive correlation of the delta connection s2–s4 (R.PCC–R.ACC) with the alpha connections s7–s12 (L.PFC–R.PHG), s8–s9 (R.PFC–L.LTC), s9–s11 (L.LTC–L.PHG), and s10–s12 (R.LTC–R.PHG); (ii) positive correlation of the delta connections s2–s8 (R.PCC –R.PFC) and s6–s8 (R.IPL–R.PFC) with the beta connection s3–s12 (L.ACC–R.PHG); (iii) positive correlation of the theta connection s6–s11 (R.IPL–L.PHG) with the beta connections s7–s11 (L.PFC–L.PHG) and s8–s9 (R.PFC–L.LTC); and (iv) negative correlation of the theta connection s8–s12 (R.PFC–R.PHG) with the gamma connection s11–s12 (L.PHG–R.PHG). 

## 4. Discussion

The goal of the study was to identify features that may accurately classify AUD individuals from unaffected controls using multi-domain measures such as EEG-based FC of the DMN, neuropsychological performance, and impulsivity in an RF classifier algorithm. Results showed that the RF model achieved 80% classification accuracy and correctly identified 24 of 30 subjects in each group. The top features that contributed to group classification (ALC vs. CTL) were: 29 FC connections (across key DMN nodes representing all frequency bands), three neuropsychological variables (total number of correctly performed trials in forward and backward sequences, and average time of correct trials in forward sequence) and all four impulsivity scores (motor, non-planning, attentional, and total). The AUD group showed a predominant pattern of hyperconnectivity among all significant connections (25 of 29 connections, *p* < 0.05) (Figure 5, panels a–e) as well as among 12 highly significant connections (11 of 12 connections, *p* ≤ 0.001) (Figure 6), where parahippocampal connections with other DMN regions were predominant (13 connections overall; six highly significant connections). On the other hand, four prefrontal connections of PFC and ACC with other right-hemispheric regions (IPL, LTC, and PHG) had hypoconnectivity and were represented in delta (three connections) and beta bands (1 connection) (Figure 5, panels a–e). Further, the AUD group also showed poor memory performance and increased impulsivity. Identified features from all three domains had significant associations with one another and with AUD status. The interpretations and implications of each of these findings are discussed below.

### 4.1. Aberrations in EEG Source DMN FC in AUD

#### 4.1.1. Predominant Pattern of DMN Hyperconnectivity in AUD

The RF algorithm identified 29 DMN connections, representing all frequency bands, as significantly important to classify AUD individuals from the unaffected controls. The predominant pattern across these DMN connections was hyperconnectivity (i.e., increased FC magnitude in 25 of 29 connections, *p* < 0.05) (Figure 5, panels a–e). Among these important 29 connections, 12 connections satisfied a higher threshold of significance (*p* ≤ 0.001) (Figure 6). Interestingly, 11 of these highly significant connections showed hyperconnectivity in AUD. It was interesting to find that this pattern of predominant hyperactivity was not restricted to any specific frequency band(s) nor any region-specific DMN seeds. In other words, the hyperactivity was a ubiquitous pattern ranging all frequencies and all 12 DMN seed regions. Broadly, these findings may indicate an aberrant network functioning during resting state in AUD individuals. It is likely that resting state hyperconnectivity across DMN connections may be suggestive of neural hyperexcitability and disinhibition in AUD individuals [108,109,110,111,112], possibly modulated by GABAergic and glutamatergic mechanisms underlying neural excitability reflected in EEG and acute and chronic effects of alcohol in the brain [113,114,115,116,117]. While neural disinhibition in other electrophysiological measures (e.g., low P3 amplitude and suppressed delta and theta oscillations underlying P3 during cognitive processing, and increased resting state beta power) have been reported [108,118,119,120], resting state EEG source FC may serve as an important and novel index of neural disinhibition in AUD and other externalizing disorders, as it is a direct measure of neural communication and brain (dys)function [57]. Since there is only a single eLORETA study on AUD [59], and it is worth comparing our findings to it. Although both studies have examined resting state EEG source connectivity in AUD using eLORETA and showed similar finding of increased FC network multiple brain regions, Huang et al. [59] derived seed regions from fMRI activations during a cue-reactivity task and reported hyperconnectivity only in theta band across reward and executive network regions. Similar findings have also been reported in other addictive behaviors. For example, hyperconnectivity across long-range connections frontal-parietal regions in theta and alpha bands have been reported in food addicted individuals [121]. Although these related studies may confirm the findings of the current study, more FC studies on AUD are required to compare methods and findings across studies to validate our results.

#### 4.1.2. Parahippocampal Hyperconnectivity in AUD

Among the 29 significant DMN connections identified by the RF model, 13 of them (~45%) were bilateral parahippocampal connections to prefrontal (PFC and ACC), temporal (LTC) and parietal (IPL) regions, and thus formed a hub region. These 13 hippocampal connections represented all frequencies (i.e., one in delta, four in theta, three in alpha, three in beta, and two in gamma band) and 12 of these connections, except the R.PHG–L.ACC beta band connection, were hyperconnected in AUD individuals. It should be mentioned that it is the parahippocampal region that links the DMN connections with the medial temporal lobe memory system [122]. Therefore, this finding of hyperconnected hippocampal hub may suggest either a noisy communication across hippocampal sub-networks or an upregulated connectivity pattern representing overcompensation for the existing neural damage and memory impairments due to chronic drinking. In our earlier study using structural MRI, Pandey et al. [16] reported smaller volume in several regions, including bilateral hippocampi, in AUD individuals compared to controls. Therefore, it is possible that damage to hippocampal connectivity to other regions of higher cortical functions (PFC, ACC, and LTC) may underlie cognitive impairments including memory deficits and blackouts that are common in chronic AUD patients [123,124,125,126]. Interestingly, using the same sample AUD subjects, we reported reduced bilateral hippocampal volume which was also associated poor visual memory performance [16]. The finding that partly supports the current findings is from the only available eLORETA based FC study on AUD [59], where the authors reported theta band hyperconnectivity in hippocampal sub-networks among the dense connections in a small sample of craving, drug-resistant, relapsed AUD individuals (N = 11). Primary involvement of theta band may also assume significance in the context of our finding that the highest number of hippocampal connections were observed in the theta band (Figure 5, panel b), while another study reported that hippocampal atrophy was correlated with EEG theta power in elderly subjects with a range of cognitive impairment [127]. Similarly, impaired hippocampal connectivity associated with memory deficits were also reported in Alzheimer’s disease [128], temporal lobe epilepsy [129,130], and elderly individuals [131]. Although EEG source FC studies on AUD are scarce, aberrations in fMRI FC across the hippocampal connections have been reported in other related conditions such as drug addiction [132]. Finally, it should also be noted that the single beta connection that showed hypoconnectivity (R.PHG–L.ACC) (Figure 5, panel d) was also found to have hyperconnectivity in the Gamma band. Although intriguing, it is possible that the reciprocal communications across hippocampal and ACC may involve different frequencies, given that these complex, multi-level connections involve both direct and indirect pathways [133]. 

#### 4.1.3. Hypoconnectivity of Prefrontal Nodes in AUD

Amidst the predominant profile of hyperconnectivity in AUD, 4 of the 29 connections across prefrontal regions (PFC and ACC) and posterior right-hemispheric regions (IPL, LTC, and PHG) showed hypoconnectivity in delta and beta band (Figure 5, panels a & d). The strongest hypoconnectivity (*p* ≤ 0.001) was observed between left and right PFC in the delta band (Figure 6). Delta rhythm consists of slow oscillations that are involved in the dynamic coordination of large-scale cortical networks and modulation of faster rhythms through cross-frequency coupling [134]. Therefore, it is possible that hypoconnectivity of these prefrontal nodes with other DMN regions observed in both slow (delta) and fast (beta) oscillations in AUD individuals may have been primarily caused by the dominant delta rhythm which may have also modulated the connectivity in beta oscillations via cross-frequency coupling. On the other hand, since the connectivity across these regions were relatively weaker in AUD (i.e., hypoconnectivity) than controls, these results may suggest a weaker or fractured connectivity of the prefrontal nodes with other cortical regions. It is well-established that the prefrontal cortex, which is highly interconnected with other cortical and subcortical regions [135], is known to mediate a range of higher-order brain functions, viz., executive functions, inhibitory control, emotional regulation, and working memory [136,137]. Therefore, a weaker prefrontal connectivity in AUD individuals may indicate higher-order cognitive deficits, which could be due to their chronic, excessive drinking resulting in disruption in the brain structure, physiology, and function [4,138]. Alcohol dependent patients have shown a variety of prefrontal lobe abnormalities [8,9], including that of executive functions [139], prefrontal volume loss [140,141] and reduced blood flow in the prefrontal areas [142,143]. In the same groups of subjects as the current study, we examined fMRI FC of the DMN and found that AUD group showed hyperconnectivity within frontal regions while showing hypoconnectivity across long-range interhemispheric and anterior-posterior connections. Although frontal hypoconnectivity in EEG and hyperconnectivity in fMRI as manifested by AUD are seemingly contradictory, it is quite possible in the context of biophysical basis of the two modalities with regard to excitation and inhibition [144,145]. Recent fMRI studies have reported restricted PCC connectivity with right medial frontal gyrus during resting state [26] as well as cue-elicited prefrontal hypoactivation in abstinent AUD individuals [146]. These findings suggest that hypoconnectivity of the prefrontal sub-network may indicate a fractured prefrontal network and associated dysfunction in executive functioning and reward/affective processing in AUD individuals [20,147]. 

### 4.2. Poor Neuropsychological Performance in AUD

The RF classifier identified three scores of memory performance in VST (i.e., total number of correctly performed trials in forward and backward sequences, and average time of correct trials in forward sequence) as important to classify AUD from CTL individuals. In other words, the AUD participants showed poor performance in visual memory capacity (forward span), visual working memory (backward span) and slower memory performance (average time taken during encoding and/or retrieval) compared to CTL participants. While the forward recall mainly involves passive storage of the items, backward recall additionally involves attentional demand [148]. The backward condition of the VST taps both immediate visual memory and components of visuospatial working memory [149]. Therefore, lower scores in forward and backward span in AUD group indicate deficits in memory storage capacity as well as visual working memory, which are possibly resulting from chronic drinking in AUD individuals [11]. Interestingly, poor memory performance (low score on forward memory span) was associated with hyperconnectivity in three delta and alpha band connections, where AUD group showed both hyperconnectivity and poor memory performance. On the other hand, faster memory performance (shorter average time) was associated with hyperconnectivity in one delta connection, where AUD individuals manifested slower memory performance and hypoconnectivity. These findings highlighted the intricate associations among neurocognitive performance, neural connectivity, and consistent impairments in AUD. While studies have shown that AUD individuals manifest impairments in multiple domains [63,64,150,151,152,153] and some of these deficits can persist even after prolonged abstinence [11], it was surprising to find that AUD individuals did not manifest significant deficits in TOLT, which measures executive functioning such as planning and problem solving [68]. Interestingly, in our previous study with the same groups of subjects, we reported that that lower volumes in prefrontal cortex and left hippocampus observed in AUD group were associated with poorer visuospatial memory performance [16]. Furthermore, the hyperconnectivity across parahippocampal hub in the current study as well as in our fMRI FC study with the same sample of individuals [54] may also be related to the deficits in visual memory in AUD subjects, possibly representing a compensatory mechanism during the memory performance. It is likely that executive deficits may have improved, as reflected in normal TOLT performance, possibly due to prolonged abstinence from drinking in majority of the AUD individuals. Future studies employing a range of neuropsychological functions on several subgroups of AUD with different length of abstinence may resolve this puzzle.

### 4.3. Heightened Impulsivity in AUD

The RF classifier identified BIS impulsivity scores as among the top significant variables to classify AUD from the CTL individuals. The AUD group showed significantly increased impulsivity compared to the controls. This finding supports the existing view that impulsivity is a core feature of substance use disorders and may result from impaired inhibitory control [154]. We have also observed that seven of the FC variables showed significant correlations with one or more BIS scores (Figure 7). Interestingly, three of the beta band connections of R.PCC with bilateral PFC and L.PHG, which showed hyperconnectivity in AUD, were also significantly associated with three of the BIS scores (except non-planning). Since beta oscillations are associated with AUD [155] and other externalizing traits [156], associations among beta band FC, impulsivity and hyperconnectivity in AUD are in line with the view that AUD is primarily a disinhibitory disorder [111] and may be related to neuronal hyperexcitability [108]. Interestingly, similar to the current findings, impulsivity factors ranked as the topmost predictors in our previous study on fMRI FC with the same set of participants [54]. Earlier studies have also drawn etiological connections among AUD, externalizing traits such as impulsivity, and neural disinhibition in the form of electrophysiological features in AUD individuals (e.g., low P3 amplitude and delta and theta oscillations underlying P3 during cognitive processing, and increased resting state beta power) [108,118,119,120]. Importantly, impulsivity was found to be associated with reduced P3 amplitude in AUD [120] and other externalizing disorders [157,158,159]. Further, recent studies have found an association of impulsivity with resting state measures of EEG power [160], EEG-based FC [161], and fMRI-based FC [3], suggesting that impulsivity in AUD may underlie specific brain networks. 

### 4.4. Associations among AUD, FC, Impulsivity, and Neurocognition

Correlations among all the top variables of FC, neuropsychological performance and impulsivity domains are shown in Figure 7. It is interesting to find that there were multi-level associations among impulsivity, neurocognition, and neural connectivity, and their intricate and consistent relationship with AUD. There were specific correlations across (i) increased impulsivity and hyperconnectivity, (ii) poor neuropsychological performance and hyperconnectivity, and (iii) increased impulsivity and poor memory performance. Furthermore, AUD individuals manifested relatively higher impulsivity and lower memory performance, in addition to aberrant neural connectivity (predominant hyperconnectivity) which were consistent with other behavioral/cognitive measures. Specifically, hyperconnectivity in three of the significant DMN connections in the beta band, the frequency which is frequently associated with externalizing disorders [155,156], were associated with increased impulsivity scores. These findings add support to the view that alcoholism can be considered as a disinhibitory disorder [109,110,111]. Although previous studies have separately shown that AUD was associated with altered rs-fMRI FC [3,161], poor neuropsychological performance [64,150], and heightened impulsivity [65,120], no previous FC studies have examined all three domains together as done in the current study. However, similar to the current study, we also conducted an rs-fMRI FC study, and identified distinct features from all three domains that are involved in the classification of AUD from CTL subjects [54]. Lastly, it may be important to note that previous studies have found associations between eLORETA connectivity and disorder-specific biomarkers such as tau concentrations in Alzheimer’s disease [162] and neurocognitive measures such as MMSE [45], suggesting that EEG source connectivity methods may become a valuable diagnostic tool to detect/predict neuropsychiatric disorders. Therefore, in order to determine potential causal links among features from various domains, future studies may also include comprehensive measures in each domain and implement sophisticated causal pathway analyses, such as multi-level path models. 

### 4.5. Potential Limitations and Suggestions for Future Research

Although the current study has yielded interesting findings by identifying important features of EEG source FC, memory performance, and impulsivity in AUD subjects as compared to unaffected controls, we like to acknowledge possible limitations or potential concerns of the current study. First, the sample includes only males, and hence the findings are generalizable only to males with AUD. It would be important for future studies to include both genders, as there are gender differences in brain functional connectivity patterns and prevalence rates of AUD [163,164,165]. Second, although we have confirmed that age per se was not significantly correlated with the significant features discriminating between AUD and control groups, it would be important for future studies to validate our findings using age-matched groups. Third, while family history of AUD has not been analyzed in the current study, it is an important variable to include in the models, and studies are underway in our lab to examine FC measures in high-risk individuals. Fourth, although eLORETA has been reported to have excellent localization accuracy as validated by several multimodal studies, caution needs to be exercised especially while considering the findings for any clinical applications; it should be kept in mind that the FC measures used in the study are the derivatives of current density sources which are in turn derived by an inverse solution employed in the eLORETA algorithm which, like other similar methods, depends on several model constraints [166]. Fifth, the RF model in the current study has not included genomic factors (e.g., specific molecular genetic variants, polygenic scores, etc.), and future studies may also explore connectome genetics [167], which is advancing on many fronts and promises to shed light on how disease risk genes affect the brain connectivity [168,169]. Sixth, although there were seemingly interesting correlations among several connections within and across frequencies (Figure 7), they were not discussed as it was beyond the scope of the current study. Seventh, although DMN is the most widely studied network, comparison across other resting state and task-related networks may further our understanding of brain dynamics in AUD. Finally, using FC measures of both EEG source and fMRI activations from the same groups of subjects will be highly useful not only to understand each method one in the light of another but also to potentially augment predictive power to classify a disorder from technically different but complementary features, and such studies are also underway in our lab.

## 5. Summary and Conclusions

In the current study, the RF model, with a classification accuracy of 80%, identified the following multi-domain features that significantly contributed to group classification (ALC vs. CTL): 29 FC connections across key DMN nodes representing all frequency bands, three neuropsychological scores representing memory performance and all four BIS impulsivity scores. The predominant pattern of FC was hyperconnectivity (25 out of 29 connections), dominated by parahippocampal connections with other regions, indicating possible neural hyperexcitability and/or compensatory mechanism. Hypoconnectivity across prefrontal nodes (bilateral PFC and L. ACC) and other right-hemispheric nodes (IPL, LTC, and PHG) suggested possible impairments in higher-order cognitive processes. Further, the AUD group also showed poor memory performance and increased impulsivity compared to CTL individuals. The top important features from all three domains had significant associations with each other as well as with AUD status. In sum, the RF model elicited important multi-domain features that significantly contributed to the classification of ALC from CTL individuals. 

The findings of the present study suggest that the connections within the DMN regions, primarily involving parahippocampal and prefrontal hub regions, are dysregulated in AUD individuals leading to possible neurocognitive deficits and increased impulsivity as observed in the current study and the previous studies. Specifically, the identification of specific connectivity patterns in discrete frequency bands in different stages of AUD may aid in diagnostic and intervention strategies based on brain circuits. These may include connectivity-guided clinical applications such as transcranial magnetic stimulation (e.g., [170]), deep brain stimulation (e.g., [171]), neurofeedback (e.g., [172]), and cognitive training [173], which have been implemented in neuropsychiatric conditions including alcohol/drug addiction and are becoming important tools for treating AUD.

## Figures and Tables

**Table 1:** Demographic and clinical characteristics of the sample. Sample size (N), mean, and standard deviation (SD) are provided.

Variable	AUD			CTL		
	N*	Mean	SD	N*	Mean	SD
**Age (in years)**	30	41.42	7.31	30	27.44	4.74
**Education (in Years)**	30	11.93	2.35	30	15.77	1.87
**Age of onset (regular alcohol use)**	30	15.77	2.58	12	20.50	3.80
**Alcohol: Drinks/day (heaviest period of alcohol use)**	30	12.08	10.02	12	2.88	1.93
**Alcohol: Days/month (heaviest period of alcohol use)**	30	20.30	9.01	12	3.35	3.64
**Alcohol: Drinks (last 6 months)**	30	2.68	6.61	18	2.61	1.98
**Alcohol: Days (last 6 months)**	30	3.97	8.02	18	2.94	3.62
**Length of abstinence from drinking (in months)**	30	22.43	28.16	18	1.9	4.99
**Tobacco: Times/day (last 6 months)**	20	9.90	5.80	6	2.33	1.63
**Tobacco: Days/month (last 6 months)**	20	28.35	4.83	6	14.17	13.82
**Marijuana: Times in last 6 months**	10	98.80	91.38	4	18.75	27.61

*N refers to the number of subjects included in these mean and standard deviation calculations for each variable. Individuals who did not consume alcohol or drugs were not included in the respective calculations.

**Table 2:** Default mode network (DMN) seed regions, Brodmann Areas (BA) and the Montreal Neurological Institute (MNI) Coordinates.

Seed	Region Name	Region Code	BA	MNI (X)	MNI (Y)	MNI (Z)
**s1**	Left posterior cingulate cortex	L.PCC	23	−10	−45	25
**s2**	Right posterior cingulate cortex	R.PCC	23	10	−45	25
**s3**	Left anterior cingulate cortex	L.ACC	32	−10	45	10
**s4**	Right anterior cingulate cortex	R.ACC	32	10	45	10
**s5**	Left inferior parietal lobule	L.IPL	40	−55	−55	20
**s6**	Right inferior parietal lobule	R.IPL	40	55	−55	20
**s7**	Left prefrontal cortex	L.PFC	46	−45	25	25
**s8**	Right prefrontal cortex	R.PFC	46	45	25	25
**s9**	Left lateral temporal cortex	L.LTC	21	−55	−15	−20
**s10**	Right lateral temporal cortex	R.LTC	21	55	−15	−20
**s11**	Left parahippocampal gyrus	L.PHG	36	−25	−30	−20
**s12**	Right parahippocampal gyrus	R.PHG	36	25	−30	−20

**Table 3:** Random Forest importance parameters (mean minimal depth, number of nodes, number of trees, times a root, accuracy decrease, Gini decrease, and *p*-value) and direction of significance for the top significant variables (*p* < 0.05) are shown. All 4 of the impulsivity scores [total, non-planning (NP), motor (MI), and attentional (AI)], 14 FC connections, and 2 neuropsychological scores from the visual span test (span and total correct scores of the forward condition) were found to be important to classify individuals into either alcohol use disorder or control group. The variables are sorted based on *p*-values.

Variable	Mean Minimal Depth	No. of Trees	No. of Nodes	Times a Root	Accuracy Decrease	Gini Decrease	*p*-Value	Direction
**BIS_Tot**	1.0667	75	75	27	0.0129	1.2864	<0.0000	Alc > Ctl
**Be_s7_s11 (L.PFC–L.PHG)**	2.1804	48	49	20	0.0052	0.6907	<0.0000	Alc > Ctl
**BIS_MI**	2.3889	46	47	18	0.0059	0.6928	<0.0000	Alc > Ctl
**BIS_NP**	2.7201	37	37	13	0.0016	0.5271	<0.0000	Alc > Ctl
**De_s7_s8 (L.PFC–R.PFC)**	2.8268	37	37	10	0.0021	0.4859	<0.0000	Ctl > Alc
**TotCor_Fw**	2.7327	34	35	17	0.0058	0.5137	<0.0000	Ctl > Alc
**Al_s9_s11 (L.LTC–L.PHG)**	2.7503	33	33	18	0.0035	0.5437	<0.0000	Alc > Ctl
**De_s2_s4 (R.PCC–R.ACC)**	3.1145	32	32	7	0.0034	0.3497	<0.0000	Alc > Ctl
**Al_s3_s7 (L.ACC–L.PFC)**	3.3615	24	25	5	0.0007	0.3068	<0.0000	Alc > Ctl
**Al_s10_s12 (R.LTC–R.PHG)**	3.3791	23	24	6	0.0023	0.2278	0.0001	Alc > Ctl
**BIS_AI**	3.3615	24	24	6	0.0022	0.2915	0.0001	Alc > Ctl
**Al_s8_s9 (R.PFC–L.LTC)**	3.3300	22	23	11	0.0015	0.2610	0.0002	Alc > Ctl
**De_s2_s8 (R.PCC–R.PFC)**	3.5566	22	22	2	−0.0006	0.2333	0.0004	Alc > Ctl
**Ga_s11_s12 (L.PHG–R.PHG)**	3.5700	22	22	3	0.0011	0.2048	0.0004	Alc > Ctl
**Be_s2_s11 (R.PCC–L.PHG)**	3.5209	21	21	5	0.0004	0.1963	0.0010	Alc > Ctl
**Be_s2_s8 (R.PCC–R.PFC)**	3.5209	21	21	7	0.0007	0.2348	0.0010	Alc > Ctl
**De_s10_s12 (R.LTC–R.PHG)**	3.5475	21	21	4	0.0004	0.2319	0.0010	Alc > Ctl
**TotCorAvgTime_Fw**	3.5251	20	20	6	0.0005	0.1813	0.0023	Alc > Ctl
**Al_s7_s12 (L.PFC–R.PHG)**	3.6802	18	19	4	0.0011	0.1598	0.0049	Alc > Ctl
**Ga_s4_s5 (R.ACC–L.IPL)**	3.6268	18	19	6	0.0001	0.2065	0.0049	Alc > Ctl
**De_s6_s8 (R.IPL–R.PFC)**	3.6226	19	19	5	0.0009	0.1804	0.0049	Ctl > Alc
**Ga_s4_s9 (R.ACC–L.LTC)**	3.5826	19	19	3	−0.0004	0.1558	0.0049	Alc > Ctl
**Al_s6_s9 (R.IPL–L.LTC)**	3.5068	18	18	9	0.0007	0.2273	0.0101	Alc > Ctl
**Th_s1_s11 (L.PCC–L.PHG)**	3.5868	18	18	4	0.0011	0.1981	0.0101	Alc > Ctl
**TotCor_Bw**	3.6177	17	17	5	0.0007	0.1670	0.0199	Ctl > Alc
**Be_s2_s7 (R.PCC–L.PFC)**	3.6044	17	17	6	0.0008	0.2282	0.0199	Alc > Ctl
**Be_s3_s12 (L.ACC–R.PHG)**	3.7644	17	17	3	0.0004	0.1473	0.0199	Ctl > Alc
**Be_s8_s9 (R.PFC–L.LTC)**	3.7377	17	17	0	−0.0005	0.1421	0.0199	Alc > Ctl
**Th_s4_s11 (R.ACC–L.PHG)**	3.5510	17	17	7	0.0006	0.2227	0.0199	Alc > Ctl
**Th_s5_s8 (L.IPL–R.PFC)**	3.5910	17	17	6	0.0001	0.1889	0.0199	Alc > Ctl
**Th_s6_s11 (R.IPL–L.PHG)**	3.7244	17	17	4	0.0002	0.1411	0.0199	Alc > Ctl
**De_s8_s10 (R.PFC–L.PCC)**	3.7953	16	16	2	−0.0009	0.1738	0.0371	Ctl > Alc
**Ga_s2_s8 (R.PCC–R.PFC)**	3.7153	16	16	3	0.0003	0.1712	0.0371	Alc > Ctl
**Ga_s3_s12 (L.ACC–R.PHG)**	3.7153	16	16	4	0.0005	0.1858	0.0371	Alc > Ctl
**Th_s2_s9 (R.PCC–L.LTC)**	3.8219	16	16	2	0.0006	0.1398	0.0371	Alc > Ctl
**Th_s8_s12 (R.PFC–R.PHG)**	3.7019	16	16	1	0.0005	0.1510	0.0371	Alc > Ctl

De–Delta; Th–Theta; Al–Alpha; Be–Beta; Ga–Gamma; L–Left; R–Right; PCC–Posterior cingulate cortex; ACC–Anterior cingulate cortex; IPL–Inferior parietal lobule; PFC–Prefrontal cortex; LTC–Lateral temporal cortex; PHG–Parahippocampal gyrus; VST–Visual Span Test [s1:s12–seeds 1-12 of the default mode network].

**Table 4:** Pearson bivariate correlations between age of the participant and the significant variables. Correlation co-efficient (*r*) and *p*-values (before Bonferroni correction) are provided for alcohol use disorder (AUD), control (CTL) group, and the total sample (ALL). None of the variables achieved the Bonferroni threshold of significance (*r* ≥ 0.49 and *p* < 0.001).

Variable	AUD		CTL		ALL^§^	
	*r*	*p*	*r*	*p*	*r*	*p*
**BIS_Tot**	0.1602	0.3977	0.0317	0.8681	0.1177	0.3746
**BIS_MI**	0.2666	0.1543	0.0642	0.7361	0.2084	0.1132
**BIS_NP**	0.0331	0.8620	0.1458	0.4422	0.0724	0.5856
**BIS_AI**	0.1106	0.5608	−0.1913	0.3113	0.0107	0.9360
**TotCor_Bw**	−0.2701	0.1489	−0.1263	0.5061	−0.2290	0.0810
**TotCor_Fw**	−0.4236	0.0197*^○^	−0.0432	0.8206	−0.2521	0.0541
**TotCorAvgTime_Fw**	−0.0204	0.9147	0.1663	0.3799	0.0360	0.7866
**Al_s10_s12 (R.LTC–R.PHG)**	0.0608	0.7495	0.1184	0.5332	0.0691	0.6032
**De_s10_s12 (R.LTC–R.PHG)**	0.1868	0.3230	−0.1175	0.5362	0.0172	0.8970
**De_s2_s4 (R.PCC–R.ACC)**	−0.2006	0.2879	−0.0383	0.8406	−0.1578	0.2328
**De_s2_s8 (R.PCC–R.PFC)**	0.0516	0.7865	−0.0467	0.8063	0.0030	0.9818
**De_s6_s8 (R.IPL–R.PFC)**	0.0688	0.7178	−0.3452	0.0617	−0.1581	0.2316
**De_s7_s8 (L.PFC–R.PFC)**	0.1907	0.3128	−0.1755	0.3535	−0.0493	0.7109
**De_s8_s10 (R.PFC–L.PCC)**	0.2100	0.2654	−0.1116	0.5572	−0.0111	0.9334
**Th_s1_s11 (L.PCC–L.PHG)**	0.1724	0.3624	0.1572	0.4067	0.1570	0.2350
**Th_s2_s9 (R.PCC–L.LTC)**	0.2825	0.1303	0.1024	0.5904	0.2351	0.0731
**Th_s4_s11 (R.ACC–L.PHG)**	0.0936	0.6226	0.0431	0.8212	0.0657	0.6213
**Th_s5_s8 (L.IPL–R.PFC)**	0.3338	0.0714	−0.0117	0.9511	0.2095	0.1113
**Th_s6_s11 (R.IPL–L.PHG)**	0.3025	0.1042	0.0510	0.7891	0.2465	0.0599
**Th_s8_s12 (R.PFC–R.PHG)**	0.1858	0.3255	−0.0576	0.7623	0.1254	0.3438
**Al_s3_s7 (L.ACC–L.PFC)**	0.0406	0.8312	−0.1670	0.3778	0.0257	0.8466
**Al_s6_s9 (R.IPL–L.LTC)**	0.0335	0.8604	0.4156	0.0224*^○^	0.0672	0.6129
**Al_s7_s12 (L.PFC–R.PHG)**	0.3021	0.1047	0.0661	0.7285	0.2500	0.0562
**Al_s8_s9 (R.PFC–L.LTC)**	−0.0817	0.6677	0.0147	0.9385	−0.0666	0.6165
**Al_s9_s11 (L.LTC–L.PHG)**	0.1162	0.5409	0.1822	0.3353	0.1168	0.3782
**Be_s2_s11 (R.PCC–L.PHG)**	0.3870	0.0346*^○^	0.3622	0.0492*^○^	0.3802	0.0030**^○^
**Be_s2_s7 (R.PCC–L.PFC)**	−0.1554	0.4122	−0.0574	0.7630	−0.1330	0.3151
**Be_s2_s8 (R.PCC–R.PFC)**	−0.1615	0.3938	0.1613	0.3946	−0.0907	0.4947
**Be_s3_s12 (L.ACC–R.PHG)**	0.3241	0.0806	0.0168	0.9298	0.1630	0.2174
**Be_s7_s11 (L.PFC–L.PHG)**	0.3038	0.1026	−0.0195	0.9185	0.2373	0.0703
**Be_s8_s9 (R.PFC–L.LTC)**	0.0397	0.8348	0.1475	0.4365	0.0708	0.5941
**Ga_s11_s12 (L.PHG–R.PHG)**	0.1716	0.3644	−0.3246	0.0801	0.0623	0.6392
**Ga_s2_s8 (R.PCC–R.PFC)**	0.1697	0.3700	−0.3420	0.0643	0.1024	0.4401
**Ga_s3_s12 (L.ACC–R.PHG)**	0.3124	0.0928	0.2271	0.2275	0.2673	0.0407*^○^
**Ga_s4_s5 (R.ACC–L.IPL)**	0.0611	0.7485	−0.2805	0.1333	−0.0137	0.9182
**Ga_s4_s9 (R.ACC–L.LTC)**	0.2004	0.2884	−0.2603	0.1647	0.1467	0.2676

Abbreviations in the functional connectivity variable labels: De–Delta; Th–Theta; Al–Alpha; Be–Beta; Ga–Gamma; s1-s12–default mode network (DMN) seeds 1-12 as listed in Table 2. [*p < 0.05; **p < 0.01; ^○^Not significant after Bonferroni correction; ^§^Based on partial correlation adjusted for group effect].

## Appendix A

Box A1: Concepts and parameters used in Random Forest classification method

**Trees:** Decision trees whose results are aggregated into one final result for classifying the factors or outcomes. Each tree is constructed based on a random (bootstrapped) subsample of the observations.
**Node:** A point in a tree, where a split occurs as a result of a ’test’ on an attribute leading to binary outcomes (e.g., whether a coin flip results in head or tail). A binary split at a node partitions the data from the parent node into two daughter nodes.
**Branch:** The outcome of the test resulting in a split or two branches in a classification tree.
**Leaf:** A terminal node that has no children or branches.
**Random Forest ensemble:** Aggregation of individual decision trees in order to combine predictions (votes) from each tree. The class/group/outcome with most votes becomes the RF model’s prediction.
**Bagging:** It’s the short form of ’bootstrap aggregating’, which is a method to improve classification by combining classifications of randomly generated training sets.
**Out of bag (OOB) estimate:** The observations that are not part of the bootstrap subsample are referred to as out-of-bag (OOB) observations. The OOB error refers to the classification error based on this subsample and serves as a validation of Random Forest model accuracy.
**Gini (mean) decrease:** It represents the importance of a specific feature/predictor/variable *(Vi)* for the classification or prediction. It’s the mean decrease in node impurity (classification error) of *Vi.* A higher Gini decrease indicates higher variable importance for *Vi*.
**Accuracy decrease:** Mean decrease in prediction accuracy after *Vi* is not taken into account.
**Mean minimal depth:** It refers to the number of nodes along the shortest path from the root node down to the nearest leaf node. Smaller depth for the *Vi* indicates its higher importance.
**Mtry:** A preset number of features/variables/predictors randomly selected (from the entire list) for splitting at each node in the construction of each decision tree.
**ntree:** A preset total number of trees to grow for a given model. Larger ‘ntree’ normally produce more stable models and more reliable predictions.
**Number of nodes:** Total number of nodes that use *Vi* for splitting (it is usually equal to number of trees if trees are shallow).
**Times a root:** Total number of trees in which *Vi* is used for splitting the root node (i.e., the whole sample is divided into two based on the value of *Vi*).
***P*-value:** Probability value of hypothesis testing based on a one-sided binomial test that indicates whether the observed number of successes (number of nodes in which *Vi* was used for splitting) exceeds the theoretical number of successes if they were random.
